# Inhibitory Activity of Synthesized Acetylated Procyanidin B_1_ Analogs against HeLa S3 Cells Proliferation

**DOI:** 10.3390/molecules19021775

**Published:** 2014-02-04

**Authors:** Syuhei Okamoto, Sayaka Ishihara, Taisuke Okamoto, Syoma Doi, Kota Harui, Yusuke Higashino, Takashi Kawasaki, Noriyuki Nakajima, Akiko Saito

**Affiliations:** 1 Graduate School of Engineering, Osaka Electro-communication University (OECU), 18-8 Hatsu-cho, Neyagawa-shi, Osaka 572-8530, Japan; E-Mails: me12a003@oecu.jp (S.O.); me12a002@oecu.jp (S.I.); me12a004@oecu.jp (T.O.); me12a008@oecu.jp (S.D.); me13a012@oecu.jp (K.H.); me13a013@oecu.jp (Y.H.); 2 Department of Pharmaceutical Sciences, Ritsumeikan University, 1-1-1 Nojihigashi, Kusatsu, Shiga 525-8577, Japan; E-Mail: kawa0227@fc.ritsumei.ac.jp; 3 Biotechnology Research Center and Department of Biotechnology, Toyama Prefectural University, 5180 Kurokawa, Imizu, Toyama 939-0398, Japan

**Keywords:** condensed tannins, oligomeric flavonoid, synthesis, cancer cells proliferation, inhibitory activity

## Abstract

Proanthocyanidins, also known as condensed tannins and/or oligomeric flavonoids, occur in many edible plants and have various interesting biological activities. Previously, we reported a synthetic method for the preparation of various procyanidins in pure form and described their biological activities. Here, we describe the synthesis of procyanidin B_1_ acetylated analogs and discuss their inhibition activities against HeLa S3 cell proliferation. Surprisingly, the lower-unit acetylated procyanidin B_1_ strongly inhibited the proliferation of HeLa S3 cells. This molecule showed much stronger inhibitory activity than did epigallocatechin-3-*O*-gallate (EGCG), green tea polyphenol, and dimeric compounds that included EGCG as a unit. This result suggests that the phenolic hydroxyl groups of the upper-units in flavan-3-ols are important for their inhibitory activity against cancer cell proliferation and that a hydrophobic lower unit dimer enhances this activity.

## 1. Introduction

There is currently a great interest in research involving compounds having strong anti-oxidation activities and superior radical scavenging abilities. Food and the ingredients that can eliminate active oxygen and free radicals have recently received increased attention. Polyphenols occur in various plants and are consumed regularly by eating vegetables and fruits. Many plants considered to be health foods have high levels of polyphenols, which are widely believed to have a beneficial impact on health. Scientific investigations of polyphenolic compounds have become increasingly important because of the various strong biological activities of these substances. For example, the flavan-3-ol EGCG (**1**), the major polyphenol in green tea, has been the focus of intense research interest because of its protective effect against a variety of cancers, such as lung, prostate, and breast [1]. In addition, surface plasmon resonance has shown the 67-kDa laminin receptor, which is widely expressed at high levels in cancer cell membranes, to be one of the receptors of EGCG [2]. This finding is evidence supporting the selective cytotoxic activity of EGCG against cancer cells. 

**Figure 1:**
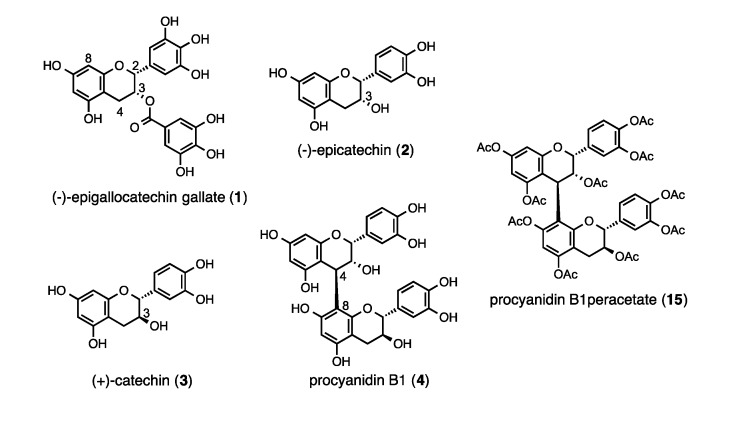
The structure of (−)-epigallocatechin gallate (**1**), (−)-epicatechin (**2**), (+)-catechin (**3**), procyanidin B1 (**4**) and procyanidin B1 peracetate (**15**).

Proanthocyanidins, which are condensed tannins or oligomeric flavonoids [3,4] are known to be extremely strong antioxidants. Research on these compounds has become increasingly important because of their various strong biological activities. However, in many cases, proanthocyanidins are obtained as a mixture of various analogs, which makes purification of each compound difficult. After an elegant contribution by Kozikowski *et al.* [5,6,7], a large number of syntheses of oligomeric catechin and epicatechin derivatives were reported [8,9,10,11,12,13,14,15]. We have also developed and reported a simple, versatile, and stereoselective methods for synthesizing procyanidin oligomers using (−)-epicatechin (**2**) and (+)-catechin (**3**) (Figure 1) [16,17,18,19,20,21,22,23,24,25,26,27,28]. In this report, we describe our synthetic methodologies for syntheses of the procyanidin B_1_ analogs **8** and **14**. We also report the surprising enhancement of the HeLa S3 cell proliferation inhibitory activity of a hydrophobic procyanidin B1 analogue in which the lower-unit was acetylated. 

## 2. Results and Discussion

**Scheme 1:**
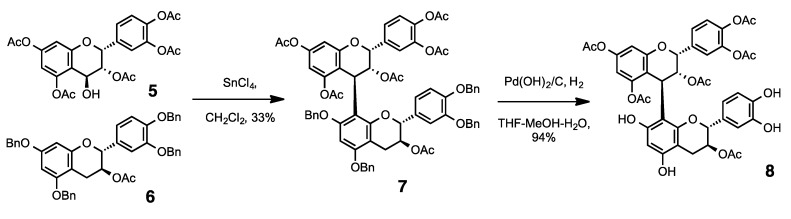
Synthesis of **8** (upper unit of procyanidin B1 is acetylated).

Our synthetic methodologies are easily applicable to various procyanidin oligomers such as the 3-*O*-substituted and partially-modified oligomers. The key step in our recently developed procyanidin synthesis method is the coupling reaction between electrophilic C-4-hydroxyl peracetates and nucleophilic compounds. As shown in Scheme 1, the electrophile **5** derived from epicatechin (**2**) was condensed with the nucleophile **6** derived from catechin (**3**) in the presence of SnCl_4_ as the Lewis acid, which produced the procyanidin B_1_ derivative (**7**) in 33% yield and with excellent stereoselectivity. Benzyl group hydrogenation of **7** was performed using H_2_/Pd(OH)_2_, which gave the upper-unit acetylated procyanidin B1 analogue **8** in good yield (Scheme 1).

**Scheme 2:**
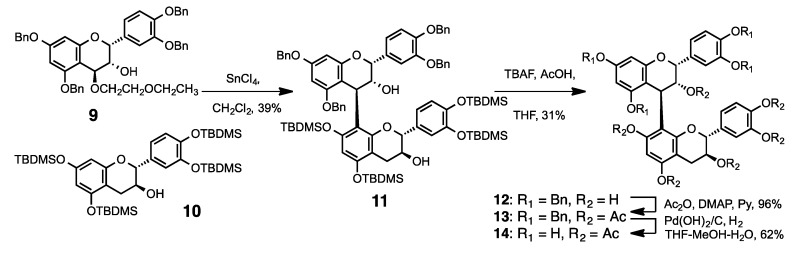
Synthesis of **14** (lower unit of procyanidin B1 is acetylated).

**Scheme 3:**
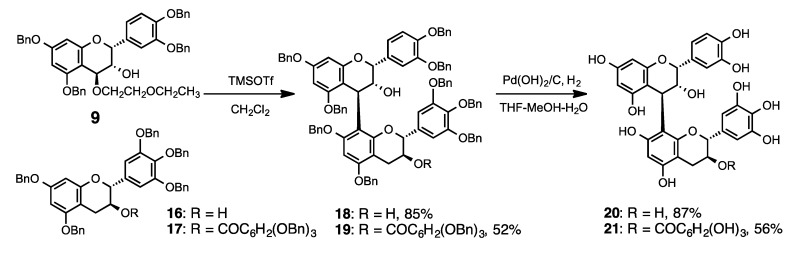
Synthesis of procyanidin B_1_ analogs.

Another general methodology for synthesizing procyanidin oligomers is shown in Scheme 2 and Scheme 3. The key step in this method is the coupling reaction between a nucleophile and a 2-ethoxyethoxy derivative of monomeric flavan-3-ol as an electrophile using a Lewis acid activator, such as TiCl_4_, SnCl_4_, or trimethylsilyl trifluoromethanesulfonate (TMSOTf). These condensation reactions proceeded smoothly and afforded good yields of 4–8 condensed oligomers with good stereoselectivity. The electrophile **9** derived from epicatechin (**2**) was condensed with the TBDMS protected nucleophile **10** [29] in the presence of SnCl_4_ as a catalyst, to produce the dimeric compound **11** in moderate yield. Deprotection of TBDMS groups by tetra-*n*-butylammonium fluoride (TBAF) followed by acetylation of phenolic groups and aliphatic hydroxyl groups gave **13**. Benzyl group hydrogenation of **13** gave the lower-unit acetylated procyanidin B1 analog (**14**). Procyanidin B1 peracetate **15** was also prepared according to a previously reported method for biological assay [28].

Furthermore, we synthesized other procyanidin B_1_ analogs that had high numbers of hydroxyl groups in their lower-units. Using the same procedure as Scheme 2, the condensation reaction between the electrophile **9** and two nucleophiles **16** or **17** produced dimeric products **18** or **19** in 85 and 52% yields, respectively. They were hydrogenated by a general procedure that gave **20** or **21** in 87% and 56% yields, respectively.

**Figure 2:**
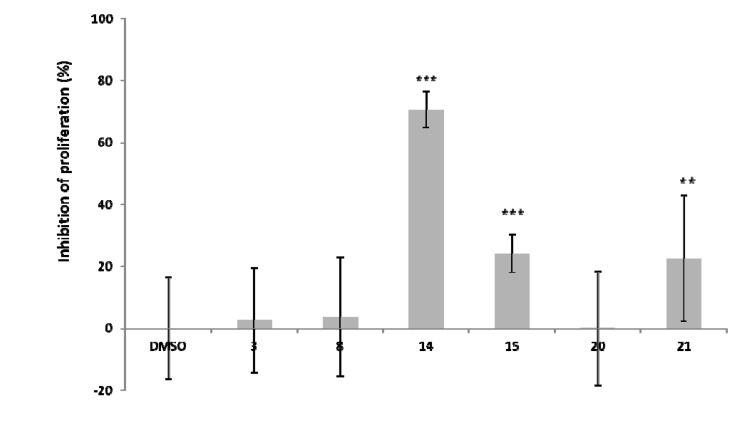
Inhibitory activity of synthetic procyanidin B_1_ analogs against HeLa S3 cell proliferation. HeLa S3 was incubated with final 100 μM of each compound for 24 h. All error bars represent standard deviations of the mean. *** *p* < 0.001, ** *p* < 0.005 *vs.* DMSO-treated groups.

The inhibitory activities of the synthetic procyanidin B_1_ analogs against HeLa S3 cell proliferation are shown in Figure 2. The inhibitory activities of epicatechin (**2**) and procyanidin B1 (**4**) were not measurable because they decomposed under the cell proliferation measurement conditions (data not shown). Compounds **2** and **4** decomposed in the medium (D-MEM) without cells and were not converted into any compounds detectable at 450 nm. The reason for this decomposition is not clear, but we think this stability of the compounds is associated with their various activities and SARs, so studies of the decomposed compounds and elucidation of the decomposition mechanism(s) are now underway. No inhibitory effect were observed for EGCG (**1**), the upper-unit acetylated procyanidin B1 **8**, and epicatechin-(4-8)-epigallocatechin (**20**). It is noteworthy that the lower-unit acetylated procyanidin B1 **14** inhibited proliferation of HeLa S3 cells quite strongly.

On the basis of these results, the upper unit of the dimeric procyanidins are considered to be the more critical part for the inhibitory activity. In addition, compounds **14** and **15**, which have hydrophobic lower-units, showed strong inhibitory activity against cell proliferation. These results suggest that the biological activities of proanthocyanidins depend not only on the number of phenolic hydroxyl groups but also on various factors such as their structures, hydrophobic properties, and hydrophilic properties. As was the case for epicatehin-(4-8)-epigallocatechin gallate (**21**), moderate inhibitory activity was observed. This activity is believed to be because of the influence of the gallic acid moiety.

## 3. Experimental

### 3.1. General

All commercially available chemicals for chemical synthesis were used without further purification. All reactions were performed under an argon atmosphere and monitored using thin-layer chromatography (TLC) with 0.25-mm pre-coated silica gel plates Merck 60F254 Art 5715. An ATAGO AP-300 spectrometer was used to measure optical rotation ^1^H-NMR spectra were recorded on a Agilent Inova 500 Spectrometer (500 MHz) and an Agilent DD2 NMR Spectrometer (400 MHz). A JEOL JMS-AX500 mass spectrometer was used to acquire fast atom bombardment (FAB) mass spectra. The human cervical adenocarcinoma cell line, HeLa-S3 (RCB0191), was provided by the RIKEN BRC through the National Bio-Resource Project of the MEXT, Saitama, Japan. Synthesized compounds were dissolved in dimethyl sulfoxide (DMSO) and stored at –25 °C.

### 3.2. Synthesis

*[4,8]-2,3-cis-3,4-trans:2'',3''-trans-3,5,7,3',4',3''-Hexa-O-acetyl-(−)-epicatechin-(+)-catechin* (**8**) A solution of **7** [28] (13.3 mg, 0.011 mmol) in THF/MeOH/H_2_O (20:1:1, 11 mL) was hydrogenated over 20% Pd(OH)_2_/C (2 mg) for 12 h at RT. Filtration and concentration afforded a pale brown solid, which was purified with preparative TLC to give 8.7 mg (0.011 mmol, 94%) of **8** as an amorphous solid. Because of NMR spectra broadening, compound **8** was acetylated according to a general procedure and then identified [28].

*[4,8]-2,3-cis-3,4-trans-2'',3''-trans-5,7,3',4'-Tetra-O-benzyl-5'',7'',3''',4'''-tetra-O-TBDMS-(−)-epicatechin-(+)-catechin* (**11**) To a solution of **9** (342 mg, 0.46 mmol) and **10** (1.35 g, 1.84 mmol) in CH_2_Cl_2_ (100 mL) SnCl_4_ (0.69 mL, 0.69 mmol, 1.0 M solution in CH_2_Cl_2_) was added dropwise in the presence of MS3Å at 0 °C. After stirring for 5 min, the pale yellow reaction mixture was quenched with saturated NaHCO_3_. The mixture was extracted with CHCl_3_ and the organic phase was washed with water and brine, and then dried (Na_2_SO_4_). Filtration, concentration, and preparative silica gel TLC purification (hexane/EtOAc, 4:1) afforded 246 mg (0.18 mmol, 39%) of **11** as a colorless amorphous substance: 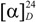 +56.4 (*c* 1.93, CHCl_3_); ^1^H-NMR (500 MHz, CDCl_3_, 0.8 : 0.2 mixture of rotational isomers) major isomer: 7.46-6.94 (18.4H, m), 6,71 (0.8H, d, *J* = 8.5 Hz), 6.49 (0.8H, d, *J* = 2.2 Hz), 6.39 (0.8H, dd, *J* = 2.2, 8.5 Hz), 6.12 (0.8H, s), 6.01 (0.8H, d, *J* = 2.2 Hz), 5.64 (0.8H, d, *J* = 2.2 Hz), 5.26–4.56 (0.8H, m), 3.96–3.95 (0.8H, m), 3.83-3.80 (0.8H, m), 3.65 (0.8H, d, *J* = 8.5 Hz), 3.67 (0.8H, dd, *J* = 6.0, 16.5 Hz), 2.52 (0.8H, dd, *J* = 8.9, 16.5 Hz), 1.70 (0.8H, d, *J* = 6.5 Hz), 1.33 (0.8H, d, *J* = 3.0 Hz), 1.04 (7.2H, s), 0.97 (7.2H, s), 0.95 (7.2H, s), 0.86 (7.2H, s), 0.30 (3H, s), 0.29 (3H, s), 0.26 (3H, s), 0.19 (3H, s), 0.16 (3H, s), 0.15 (3H, s), 0.04 (3H, s), 0.02 (3H, s); minor isomer: 7.46–6.94 (4.8H, m), 6.78 (0.2H, d, *J* = 2.2 Hz), 6.65 (0.2H, d, *J* = 2.2 Hz), 6.29 (0.2H, d, *J* = 2.2 Hz), 6.20 (0.2H, d, *J* = 2.2 Hz), 5.96 (0.2H, s), 5.33–4.54 (2H, m), 3.95–3.98 (0.2H, m), 3.89–3.88 (0.2H, m), 3.63–3.61 (0.2H, m), 3.57–3.50 (0.2H, m), 3.09–3.04 (0.2H, m), 2.62 (0.2H, dd, *J* = 9.4, 16.5 Hz), 1.65–1.50 (0.2H, m), 1.33–1.25 (0.2H, m), 1.07-0.75 (7.2H, m), 0.30–0.01 (4.8H, m); ^13^C-NMR (125 MHz, CDCl_3_, 0.8: 0.2 mixture of rotational isomers) major isomer: 158.0, 157.0, 155.4, 154.8, 152.9, 152.7, 149.0, 148.5, 147.0, 146.6, 137.54, 137.52, 137.48, 137.37, 132.9, 130.0, 128.7-126.8 (C×7), 120.8, 119.8, 119.3, 115.1, 113.8, 113.4, 106.2, 104.4, 102.2, 94.1, 93.0, 81.2, 75.3, 72.1, 71.50, 71.49, 69.7, 69.0, 68.4, 36.1, 28.9, 25.95, 25.91, 25.89, 25.81, 18.42, 18.40, 18.31, 18.29, −3.7–4.6 (C×4); minor isomer: 158.53, 158.46, 155.2, 154.7, 153.6, 152.4, 148.7, 148.1, 147.10, 147.07, 137.9, 137.7, 137.6, 137.3, 132.6, 130.8, 128.7–126.8 (C×7), 120.1, 119.1, 118.9, 115.0, 114.2, 112.0, 104.8, 104.5, 102.2, 95.0, 93.6, 81.3, 76.0, 72.5, 71.0, 70.6, 70.2, 69.6, 68.8, 36.7, 28.5, 26.4, 25.1–25.8 (Cx3), 19.1-18.0 (C×4), −3.7–4.2 (C×4); FABMS (*m/z*) 1398 (31), 1397 (43), 1396 ([M+H]^+^, 53), 838 (28), 837 (38), 836 (57), 725 (26), 722 (19), 721 (28), 654 (55), 653 (100); FABHRMS calcd for C_82_H_107_O_12_Si_4 _[M+H]^+^, 1395.6840; found: 1395.6913.

*[4,8]-2,3-cis-3,4-trans-2'',3''-trans-3,3'',5'',7'',3''',4'''-Hexa-O-acetyl-,5,7,3'4'-tetra-O-benzy-(−)-epicatechin-(+)-catechin* (**13**) A solution of 11 (246 mg, 0.18 mmol) in THF (50 mL) was added dropwise to TBAF (1.44 mL, 1.44 mmol, 1M solution in THF) in the presence of AcOH (0.08 mL, 1.44 mmol) at 0 °C. Concentration and preparative silica gel TLC purification (hexane/EtOAc, 1:2) afforded 52 mg (0.055 mmol, 31%) of **12** as a colorless amorphous substance. Compound **12** was immediately dissolved in dry pyridine (2 mL) and acetic anhydride (40 mg, 0.39 mmol) was added in the presence of cat. DMAP. The mixture was extracted with CHCl_3_ and the organic phase was washed with water and brine, and then dried (Na_2_SO_4_). Filtration, concentration, and preparative silica gel TLC purification (hexane/EtOAc, 3:1) afforded 63 mg (0.053 mmol, 96%) of **13** as a colorless amorphous substance: 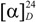 + 61.9 (*c* 0.63, CHCl_3_); ^1^H-NMR (500 MHz, CDCl_3_) 7.47–6.83 (22H, m), 7.08 (1H, d, *J* = 2.2 Hz), 7.01 (1H, d, *J* = 8.5 Hz), 6.96 (1H, d, *H* = 2.2 Hz), 6.81 (1H, dd, *J* = 2.2, 8.5 Hz), 6.46 (1H, s), 6.33 (1H, d, *J* = 2.2 Hz), 6.25 (1H, d, *J* = 2.2 Hz), 5.27 (2H, s), 5.26 (2H, s), 5.11–4.79 (9H, m), 4.95 (1H, d, *J* = 8.0 Hz), 4.77 (1H, d, *J* = 10.0 Hz), 4.71 (1H, d, *J* = 10.0 Hz), 4.47 (1H, br s), 2.99 (1H, dd, *J* = 5.5, 16.5 Hz), 2.60 (1H, dd, *J* = 8.5, 16.5 Hz), 2.33 (3H, s), 2.24 (3H, s), 2.22 (3H, s), 1.93 (3H, s), 1.57 (3H, 2), 1.52 (3H, s); ^13^C-NMR (125 MHz, CDCl_3_) 170, 168.9, 168.8, 168.7, 168.0, 167.9, 159.0, 158.0, 155.2, 152.2, 149.0, 148.7, 147.8, 147.5, 141.9, 141.6, 137.30, 137.28, 137.0, 136.6, 136.1, 131.1, 128.6-127.4 (Cx13), 124.6, 123.3, 122.3, 119.8, 119.1, 114.8, 113.5, 110.5, 102.4, 94.7, 93.6, 77.8, 77.2, 74.9, 71.5, 71.3, 70.2, 70.0, 68.3, 34.0, 25.1, 20.89, 20.87, 20.7, 20.6, 20.3, 19.9. FABMS (*m/z*) 1192 (32), 1191 ([M+H]^+^, 44), 1133 (17), 1132 (41), 1131 (66), 1130 (53), 1042 (37), 1041 (62), 1040 (82), 950 (18), 949 (28), 605 (23), 604 (45), 603 (100); FABHRMS calcd for C_70_H_63_O_18 _[M+H]^+^, 1191.4014; found: 1191.3949.

*[4,8]-2,3-cis-3,4-trans-2'',3''-trans-3,3'',5'',7'',3''',4'''-Hexaacetyl-(−)-epicatechin-(+)-catechin* (**14**) A solution of **13** (63 mg, 0.053 mmol) in THF/MeOH/H_2_O (11 mL, 20:1:1) was hydrogenated over 20% Pd(OH)_2_/C (2 mg) for 6 h at RT Filtration and concentration afforded a pale brown solid, which was acetylated according to a general procedure to give 27 mg (0.033 mmol, 62%) of **14** as an amorphous solid: 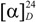 0 (*c* 0.012, CHCl_3_); ^1^H-NMR (500 MHz, CDCl_3_, 0.7:0.3 mixture of rotational isomers) major isomer: 7.25–6.41 (4.2H, m), 6.12–6.10 (1.4H, m), 6.12 (0.7H, s), 5.19–5.13 (1.4H, m), 5.02 (0.7H, d, *H* = 10.0 Hz), 4.70 (0.7H, t, *J* = 10.0 Hz), 4.43 (0.7H, br s), 2.92 (0.7H, dd, *J* = 5.0, 16.0 Hz), 2.67 (0.7H, dd, *J* = 10.0, 16.0 Hz), 2.33 (2.1H, s), 2.23 (2.1H, s), 2.21 (2.1H. s), 1.92 (2.1H. s), 1.71 (2.1H, s), 1.62 (2.1H, s); minor isomer: 7.25–6.08 (2.7H, m), 5.19–4.68 (1.5H, m), 4.47 (0.3H, br s), 2.85–2.81 (0.3H, m), 2.75–2.61 (0.3H, m), 2.32 (1.8H, s), 2.01 (0.9H, s), 1.90 (0.9H, s), 1.69 (0.9H, s), 1.65(0.9H, s); ^13^C-NMR (125 MHz, CDCl_3_) major isomer: 176.1, 174.3, 173.4, 173.2, 173.0, 173.4, 162.2, 161.4, 159.4, 159.3, 151.9, 151.7, 148.7 (×2), 140.8, 133.4, 132.9, 131.9-131.4 (×7), 123.0, 122.9, 121.6, 118.4, 117.3 (×2), 114.6, 109.0, 104.7, 99.4, 98.6, 78.5, 76.3, 73.7, 72.4, 38.0, 27.8, 23.4, 23.12, 23.05 (×2), 22.8, 22.6. Minor isomer was not identified. FABMS (*m/z*) 832 (3.6), 831 ([M+H]^+^, 9.1), 605 (27), 604 (45), 603 (100); FABHRMS calcd for C_42_H_39_O_18 _[M+H]^+^, 831.2136; found: 831.2116.

*[4,8]-2,3-cis-3,4-trans-2'',3''-cis-5,7,3',4',5'',7'',2''',3''',4'''-Nona-O-benzyl-(−)-epicatechin-(−)-epi-gallocatechin* (**18**) To a solution of **9** (39 mg, 0.053 mmol) and **16** (160 g, 0.21 mmol) in CH_2_Cl_2_ (100 mL) TMSOTf (0.13 mL, 0.064 mmol, 0.5 M solution in CH_2_Cl_2_) was added dropwise at −20 °C. After stirring for 5 min, the pale yellow reaction mixture was quenched with saturated NaHCO_3_. The mixture was extracted with CHCl_3_ and the organic phase was washed with water and brine, and then dried (Na_2_SO_4_). Filtration, concentration, and preparative silica gel TLC purification (hexane/EtOAc, 3:1) afforded 63 mg (0.045 mmol, 85%) of **18** as a colorless amorphous substance: 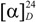 0 (*c* 0.030, CHCl_3_); ^1^H NMR (500 MHz, CDCl_3_, 0.7:0.3 mixture of rotational isomers) major isomer: 7.51-6.80 (32.9H, m), 6.45 (1.4H, s), 6.40 (0.7H, s), 6.07 (0.7H, d, *J* = 2.0 Hz), 5.75 (0.7H, d, *J* = 2.0 Hz), 5.61 (0.7H, s), 5.19–4.56 (12.6H, m), 4.71 (0.7H, d, *J* = 11.0 Hz), 4.56 (0.7H, d, *J* = 11.0 Hz), 4.12 (0.7H, br s), 4.04 (0.7H, s), 3.92 (0.7H, d, *J* = 4.5 Hz), 3.04 (0.7H, d, *J* = 17.5 Hz), 2.95 (0.7H, dd, *J* = 5.0, 17.5 Hz), 1.85 (0.7H, br s), 1.60 (0.7H, br s); minor isomer: 7.51–6.80 (13.8H, m), 6.34 (0.3H, s), 6.29 (0.3H, d, *J* = 2.5 Hz), 6.24 (0.3H, s), 6.12 (0.3H, d, *J* = 2.2 Hz), 5.19–3.95 (6.0H, m), 4.68 (0.3H, d, *J* = 12.0 Hz), 4.42 (0.3H, d, *J* = 12.0 Hz), 3.20–3.12 (0.3H, m), 3.05–3.00 (0.3H, m), 1.80–1.40 (0.6H, m); ^13^C-NMR (125 MHz, CDCl_3_, 0.7:0.3 mixture of rotational isomers) major isomer: 158.3, 157.0, 156.6, 156.9, 155.2, 154.4, 152.9, 152.5, 149.3, 148.8, 138.4–136.9 (C×11), 133.3, 132.7, 128.7–126.6 (C×21), 119.8, 115.2, 113.5, 111.3, 105.4, 104.4, 102.4, 93.8, 93.3, 91.7, 79.2, 75.7, 75.2, 72.5, 71.4, 71.3, 71.1, 70.5, 70.1, 70.0, 69.1, 66.4, 35.8, 28.6; minor isomer: 158.4, 156.9, 156.7, 155.6, 155.2, 153.1, 152.8, 149.1, 148.7, 138.4–136.9 (C×12), 133.6, 132.5, 128.7–126.6 (C×21), 120.1, 115.1, 114.2, 111.7, 105.6, 104.5, 101.8, 94.5, 93.5, 93.1, 78.3, 75.6, 75.2, 72.2, 71.6, 71.4, 71.3, 70.6, 70.1, 69.9, 69.6, 65.2, 36.1, 28.9.

*[4,8]-2,3-cis-3,4-trans-2'',3''-cis-5,7,3',4',5'',7'',2''',3''',4'''-Nona-O-benzyl-(−)-epicatechin-(−)-epi-gallocatechin-3''-O-(tri-O-benzyl)gallate* (**19**). To a solution of **9** (284 mg, 0.38 mmol) and **17** (1.79 g, 1.52 mmol) in CH_2_Cl_2_ (100 mL) was added dropwise TMSOTf (0.46 mL, 0.46 mmol, 1.0 M solution in CH_2_Cl_2_) at −20 °C. After stirring for 5 min, the pale yellow reaction mixture was quenched with sat. NaHCO_3_. The mixture was extracted with CHCl_3_ and the organic phase was washed with water and brine, and dried (Na_2_SO_4_). Filtration, concentration, and preparative silica gel TLC purification (hexane/EtOAc, 3:1) afforded 359 mg (0.20 mmol, 52%) of **19** as a colorless amorphous solid: 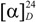 −4.6 (*c* 1.64, CHCl_3_); ^1^H-NMR (500 MHz, CDCl_3_) major isomer: 7.45–6.05 (67H, m), 5.63–5.62 (3H, m), 5.51 (1H, d, *J* = 6.0 Hz), 5.30–4.51 (26H, m), 4.14–4.10 (2H, m), 3.20 (1H, dd, *J* = 6.0, 18.5 Hz), 3.04 (1H, d, *J* = 18.5 Hz), 1.78 (1H, d, *J* = 6.0 Hz); ^13^C-NMR (125 MHz, CDCl_3_) 165.3, 158.4, 156.9, 156.2, 155.4, 154.8, 152.8, 152.5, 149.3, 148.9, 143.4, 138.1–136.5 (C×7), 132.8, 132.3, 128.7–125.7 (C×26), 119.7, 114.5, 113.6, 111.9, 109.9, 106.0, 105.8, 104.2, 102.6, 93.8, 93.4, 91.8, 78.4, 75.9, 75.3, 75.1, 75.1, 71.8, 71.4, 71.2, 70.8, 70.7, 70.2, 69.2, 68.9, 35.9, 26.9.

*[4,8]-2,3-cis-3,4-trans-2'',3''-cis-(−)-Epicatechin-(−)-epigallocatechin* (**20**). A solution of **18** (63 mg, 0.045 mmol) in THF/MeOH/H_2_O (20:1:1, 22 mL) was hydrogenated over 20% Pd(OH)_2_/C (2 mg) for 12 h at RT. Filtration and concentration afforded a pale brown solid, which was purified using HPLC to give 38 mg (0.039 mmol, 87%) of **20** as an amorphous solid. 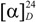 −12.5 (*c* 0.016, CH_3_OH); ^1^H NMR (400 MHz, CD_3_OD, −40 °C) 6.82 (1H, br s), 6.87 (1H, d, *J* = 8.5 Hz), 6.59 (1H, br d, *J* = 8.5 Hz), 6.59 (2H, s), 5.86 (1H, s), 6.10–5.15 (2H, m), 5.03 (1H, br s), 4.86 (1H, br s), 4.60 (1H, br s), 4.25 (1H, br s), 3.79 (1H, br s), 2.92 (1H, br d, *J* = 16.0 Hz), 2.79 (1H, d, *J* = 16.0 Hz); FABMS (*m/z*) 596 (29), 595 ([M+H]^+^, 27), 581 (33), 561 (18), 537 (26), 493 (26), 329 (100); FABHRMS calcd for C_30_H_27_O_13 _[M+H]^+^, 595.1452; found: 595.1402.

*[4,8]-2,3-cis-3,4-trans-2’’,3’’-cis-(−)-Epicatechin-(−)-epigallocatechin-3''-O-gallate* (21) A solution of **19** (164 mg, 0.090 mmol) in THF/MeOH/H_2_O (20:1:1, 22 mL) was hydrogenated over 20% Pd(OH)_2_/C (2 mg) for 12 h at RT. Filtration and concentration afforded a pale brown solid, which was purified using HPLC to give 38 mg (0.051 mmol, 56%) of **21** as an amorphous solid. 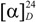 0 (*c* 0.025, CH_3_OH); ^1^H NMR (400 MHz, CD_3_OD, −40°C) 7.01 (2H, s), 6.89 (1H, br s), 6.87 (1H, br s), 6.80 (6.60 (2H, m), 6.58 (2H, s), 6.13 (1H, br s), 5.89 (1H, br s), 5.55 (1H, br s), 5.14 (1H, br s), 5.12 (1H, br s), 4.78 (1H, br s), 4.54 (1H, br s), 3.84 (1H, br s), 3.14–2.72 (2H, m); FABMS (*m/z*) 749 (18), 748 (25), 747 (27), 746 ([M+H]^+^, 17), 719 (17), 603 (21), 545 (23), 503 (20), 487 (23), 329 (100); FABHRMS calcd for C_37_H_31_O_17 _[M+H]^+^, 747.1561; found: 747.1519.

### 3.3. Inhibitory Activity of Cell Proliferation

HeLa S3 cells were seeded into a 96-well flat-bottomed tissue culture plate (Iwaki) in a 37 °C incubator equilibrated with a 5% CO_2_: 95% humidified air atmosphere. D-MEM (Dulbecco's Modified Eagle’s Medium; Gibco^®^ (Life technologies, Grand Island, NY, USA) supplemented with 5% fetal calf serum and 1% Pen-Strep; Invitrogen^TM^ (Life technologies, Grand Island, NY, USA). After 24 h of incubation, synthesized compounds in DMSO were added (final 100 μM) and incubated for 24 h. A Cell Count Reagent SF colorimetric assay (Nacalai Tesque, Kyoto, Japan) was performed to evaluate the inhibitory activity of cell proliferation. In brief, 10 µL of Cell Count Reagent SF was added to each well, and incubation was performd for 2 h at 37 °C. Following which, viable cells were assessed using a microplate reader (Filter Max F5 multi-mode microplate reader; Molecular Devices, Downingtown, PA, USA) to measure the OD at 450 nm. Cells cultured with DMSO treatment served as the control.

## 4. Conclusions

In conclusion, we have used our synthetic methodologies to synthesize various procyanidin B_1_ analogs to clarify the biological activities of the compounds. Investigation of cell proliferation inhibitory activity against HeLa S3 cells showed that free-phenolic hydroxyl groups were important for the activity and that lower-unit hydrophobicity enhanced this activity. Biological and chemical mechanistic studies are currently being conducted.
